# The Role of Ataxia Telangiectasia Mutant and Rad3-Related DNA Damage Response in Pathogenesis of Human Papillomavirus

**DOI:** 10.3390/pathogens9060506

**Published:** 2020-06-23

**Authors:** Ying Luo, Shiyuan Hong

**Affiliations:** Institute of Life Sciences, Chongqing Medical University, Chongqing 400016, China; luoyingcqmu@163.com

**Keywords:** Human papillomavirus (HPV), life cycle, HPV DNA replication, genome amplification, DNA damage response (DDR), the ATR pathway, cervical cancer

## Abstract

Human papillomavirus (HPV) infection leads to a variety of benign lesions and malignant tumors such as cervical cancer and head and neck squamous cell carcinoma. Several HPV vaccines have been developed that can help to prevent cervical carcinoma, but these vaccines are only effective in individuals with no prior HPV infection. Thus, it is still important to understand the HPV life cycle and in particular the association of HPV with human pathogenesis. HPV production requires activation of the DNA damage response (DDR), which is a complex signaling network composed of multiple sensors, mediators, transducers, and effectors that safeguard cellular DNAs to maintain the host genome integrity. In this review, we focus on the roles of the ataxia telangiectasia mutant and Rad3-related (ATR) DNA damage response in HPV DNA replication. HPV can induce ATR expression and activate the ATR pathway. Inhibition of the ATR pathway results in suppression of HPV genome maintenance and amplification. The mechanisms underlying this could be through various molecular pathways such as checkpoint signaling and transcriptional regulation. In light of these findings, other downstream mechanisms of the ATR pathway need to be further investigated for better understanding HPV pathogenesis and developing novel ATR DDR-related inhibitors against HPV infection.

## 1. Introduction

Human papillomavirus (HPV) is a small double-stranded DNA virus that is the etiological cause of many human cancers including cervical, anal, vaginal, vulvar, and penile, as well as oropharyngeal cancers [1]. Among them, cervical cancer is the fourth most common deadly cancer for women worldwide. In 2018, about 570,000 incidences of cervical cancer were reported and 311,000 related deaths occurred [2]. Although screening techniques and vaccination greatly protect people from HPV-related diseases such as cervical cancer, anal cancer and genital warts [3,4], there are no clinical drugs for patients that have been infected. Thus, it is still important to better understand HPV pathogenesis by further investigating molecular mechanisms of the HPV life cycle.

The key events involved in the differentiation-dependent HPV viral life cycle include viral entry, viral replication, and virion formation as well as release. HPV replication is dependent on host cellular mechanisms including cell cycle, apoptosis, transcriptional regulation, DNA damage response (DDR), etc. Among them, DDR is a complex signaling network that occurs naturally in cells to prevent the replication mechanism from dysfunction in response to changes in the structure of genetic material [5]. The ataxia telangiectasia mutant and Rad3-related kinase (ATR) serves as a key DDR kinase to respond to single-strand or double-strand breaks (SSBs or DSBs). When replication stress occurs, activated ATR phosphorylates many substrates and consequently contributes to DNA replication, cell mitosis, apoptosis, etc. [6]. In absence of external DNA damage signals, HPV induces activation of the ATR pathway. Inhibition of ATR activation leads to a suppression of HPV genome maintenance [7] and amplification [8]. Here, we will briefly review how HPV manipulates DDR in host cells for efficient viral DNA replication, and mainly discuss the essential role of the ATR pathway for the HPV life cycle. In addition, we will summarize the current findings of ATR-related small-molecule inhibitors [9,10] in HPV-related tumors.

## 2. Epidemiology and Current Prophylactic Strategies against HPV Infection

### 2.1. Epidemiological Characteristics

More than 170 HPV genotypes have been reported [11]. These viruses mainly comprise five genera, including α, β, γ, μ, and ν. HPV often infects the cutaneous or mucosal tissues [12]. The members of the α papillomaviruses family are divided into low-risk and high-risk types (HPV16, 18, 31, 33, 35, 39, 45, 51, 52, 56, 58, 59, and 68) according to their association with cancers [13]. Low-risk types are mainly related to genital papilloma, warts, or other benign lesions, while high-risk ones lead to cervical cancer and other cancer types described above [14]. Cervical cancer is the fourth most common cause of cancer incidence and mortality among women worldwide, and still the leading risk factor of cancer-related death for women in developing countries [2]. Anal cancer is another HPV-related cancer mainly associated with HPV16 infection, which occurs more commonly in women than in men [15]. Squamous intraepithelial lesions (SIL), such as low-grade SIL (LSIL) and high-grade ones (HSIL), are precancerous squamous cells on the surface of the cervix, anus, vulva, and vagina. They are also usually caused by HPV infection [12]. Additionally, HPV-positive head and neck squamous cell carcinoma (HNSCC) has recently become prevalent especially in Europe and North America [16]. To protect people from cervical cancer, several vaccines have been developed in addition to the use of preventative screening. 

### 2.2. Cervical Cancer Screening

The American Society of Clinical Oncology recommends that cervical cancer screening is carried out among women of ages from 30 to 49 in a timely manner [17]. At present, there are mainly three techniques: cytologic testing (recommended for age 21 to 29), combination with cytology and HPV testing (for age 30 to 65), and high-risk HPV testing (targeting HPV16, 18 and other high-risk subtypes for women age 25 and above) [3,18,19]. As an early method, cytological screening has certain benefits [3], but is limited by low sensitivity and poor consistency. The combinational detection comes with an age limitation because HPV testing is not appropriate for women younger than 30 [20]. High-risk HPV testing outbids the previous two for its high sensitivity, great reproducibility, and easy quality control [18,19]. However, the latter loads a financial burden and lacks coverage for many other HPV types, which raises a potential issue that might be solved by vaccines covering more HPV subtypes. 

### 2.3. The Pros and Cons of the HPV Vaccines against Cancer

Three generations of cervical cancer vaccines have been developed within 10 years [4,21]. The first bivalent HPV vaccine (Cervarix), targeting HPV16 and HPV18, protects adolescents and young women from age 10 to 25 [22]. The later quadrivalent HPV vaccine (Gardasil) against HPV6, HPV11, HPV16, and HPV18 can be applied for adolescents and young adults aged 9 through 26 years [23]. In addition to those 4 types, the nine-valent vaccine (Gardasil 9) prevents infection from HPV31, HPV33, HPV45, HPV52, and HPV58 [4]. These vaccines also greatly protect HPV-uninfected girls or young women from benign lesions such as anal genital warts (90% of which are caused by HPV6 and HPV11) [24]. In addition, the vaccines also benefit men for prevention of precancerous lesions and related cancers [25]. Currently, scientists continue to develop newly multi-valent vaccines covering more HPV subtypes.

While they are approved for prevention of genital warts and cervical cancer, HPV vaccines encounter challenges in clinical application [26]. One major issue is that women younger than 25 years who have engaged in sex are not recommended to be vaccinated. A portion of allergy safety issues during vaccination have arisen, leading to an increase of mistrust in population. The financial cost for the vaccines is still a big concern for low-income families. It is not well defined yet whether the vaccines are effective through our lifetime. Finally, these vaccines can only protect against, but are not effective in treating, existing HPV infections. Anti-HPV therapeutic vaccines are at various stages of clinical development for HPV-related precancers, but are not yet approved [27].

## 3. HPV Life Cycle

### 3.1. The Structure of HPV and the Function of Viral Proteins

**Figure 1:**
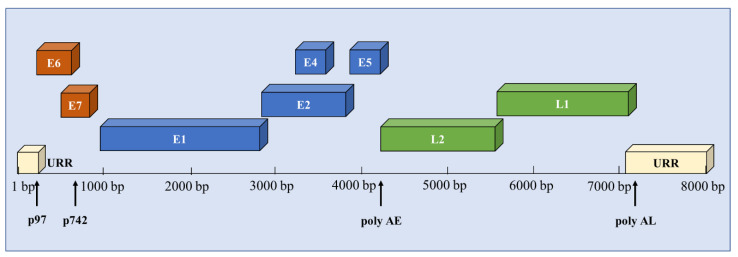
The schematic HPV 31 genome structure. The linearized HPV 31 genome is 8000 bp in size and encodes gene products in three regions: the early, late, and upstream regulatory regions (URR). The genes on the early region encode E1, E2, E4, E5, E6, and E7. The late genes represent capsid proteins L1 and L2. The URR contains the replication starting point and transcription factor binding sites. HPV31 has two well-characterized promoter elements known as early promoter (p97) and late promoter (p742). poly AE and poly AL indicate the positions of polyadenylation early and late sites shown as arrows.

The HPV virion is a nonenveloped icosahedron with a diameter of about 55 nm. The 8 kb circular double-stranded DNA genome consists of three regions: the early, late, and upstream regulatory region (URR) [28], as shown in Figure 1. The genes located on the early region (E1, E2, E4, E5, E6, and E7) are indispensable components of the virus life cycle. The late genes represent capsid proteins L1 and L2. The URR regulates the transcription of viral genes and contains the replication starting point as well as transcription factor binding sites. The expression of viral genes can be initiated by both the early and late promoters. The early promoters (p97) can guide the transcription of early viral proteins, for maintaining the stability of the virus genome. The late promoters (p742 in HPV31 or p670 in HPV16) are activated during epithelial differentiation to regulate the late gene expression, genome amplification, and viral production.

HPV proteins play diverse roles in the HPV life cycle. For example, as a DNA helicase/ATPase, E1 protein is required throughout progression of HPV DNA replication [29]. E1 also recruits host DNA polymerases to viral origins and interacts with E2 to initiate HPV DNA replication [30,31]. E2 possesses DNA-binding domains to tether the viral genome to chromosomes and has the capacity of regulating the HPV early promoter [31,32]. Considered as an important marker of active HPV infection, E4 contributes to HPV DNA replication, capsid assembly, and disruption of epithelial cell intermediate filament network [33,34,35]. Expression of E5 is necessary for HPV late gene expression and genome amplification [36]. In addition, E5 can alter expression of many genes involved in cell motility, adhesion, and proliferation [37,38]. The high-risk E6 degrades p53 through binding to the cellular E3 ubiquitin ligase E6-associated protein (E6AP) to block apoptosis [39]. E7 binds and inactivates retinoblastoma tumor suppression protein (pRB), leading to malignant cell cycle progression [40,41]. L1 and L2 contribute to virion assembly as well as viral chromatin packaging [42,43].

### 3.2. The Life Cycle of HPV

**Figure 2:**
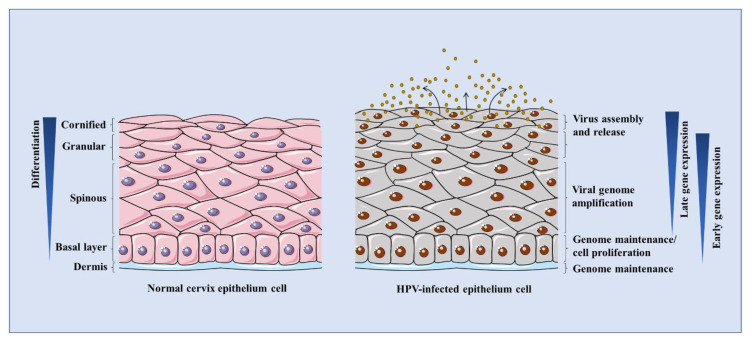
The schematic illustration of the HPV life cycle. Epithelial differentiation processes are compared between normal and HPV-infected keratinocytes. HPVs infect keratinocytes in the basal layer when wounded. Early genes E1, E2, E6, E7 are expressed at low levels for genome maintenance and cell transformation. The viral genome is maintained at the basal layer. Upon differentiation, HPV late genes such as E1, E4^E5, L1, and L2 are expressed. The viral genomes are greatly amplified, assembled, and newly synthesized virions are released from the uppermost layers of epithelium.

HPV infects the epithelial basal layer possibly through micro-wounds. The infected cells proliferate to construct a base pool for papilloma. HPV episomes are delivered into the host nucleus to cause an infection [12]. During this process, the infected cells are prevented from exiting the cell cycle. Meanwhile, HPV utilizes the cellular contents to accomplish its own replication in synchrony with host cellular DNA at a low-copy rate [44]. After mitosis, the infected daughter cells leave the basal layer and are pushed towards the stratified layers. When the late promoter of HPV is activated by differentiation signals, the viral genomes are replicated to thousands of copies per cell in the process of vegetative genome replication (so referred as to amplification) [45]. The capsids are then synthesized and virions are assembled in the upper terminally differentiated layers [46]. Eventually, the mature viral progeny is released from the uppermost layers of the epithelium, as shown in Figure 2.

### 3.3. Mechanisms of HPV Life Cycle

To accomplish its life cycle, HPV regulates host cellular mechanisms such as cell cycle, transcription regulation, DDR, etc. Among them, cell cycle mechanisms have been extensively studied. For example, HPV E6 induces phosphorylation of signal transducer and activator of transcription protein-3 (STAT-3) to enhance cyclin D1-mediated cell cycle progression and to facilitate HPV genome amplification [47]. HPV16 E7 blocks p21’s inhibition of cyclin-dependent kinase (CDK) activities and proliferating cell nuclear antigen (PCNA)-dependent DNA replication [48]. HPV E7 also promotes cell cycle progression by increasing cyclin A and cyclin E dependent CDK2 complex activity [49,50] or activating E2F transcription factors in a pRB-independent manner [51,52].

Transcription regulation plays key roles in HPV productive life cycle. For example, the transcription factor family of p63 and p73 is critical for HPV genome amplification [53,54]. Other transcription factors that regulate HPV viral transcription were summarized in the previous review [55]. Among them, YY-1 (Yin and Yang 1), TBP (TATA-box-binding protein), AP-1 (Activator protein 1), Sp1, and Oct-1 (Octamer-binding protein 1) act on HPV early promoter, while C/EBPβ isoforms LIP and LAP work on the late promoter. YY-1, Sp1, and Oct-1 are also associated with DDR [56,57,58]. Members of STAT proteins such as STAT-1 and STAT-5 participate in regulation of HPV genome maintenance and amplification [59,60]. STAT-1 is suppressed by HPV and this suppression facilitates HPV genome amplification [59]. In contrast, STAT-5 and its associated transcription factor Kruppel-like factor13 can promote the ataxia-telangiectasia mutated (ATM) DDR signaling to contribute to HPV replication [60,61]. Furthermore, STAT-5 can induce the ATR DDR to regulate HPV genome maintenance and genome amplification [8]. In addition, altered expression of microRNAs (miRNAs) has been identified as biomarkers of high-risk HPVs infection [62]. Previous studies found that over-expression of miR-203, miR-145, or miR-125b leads to a reduction of HPV genome amplification or gene expression [63,64,65]. 

Other than cell cycle and transcriptional regulation, HPV DNA replication requires activation of many DDR pathways including the ATR signaling, which we will introduce in a separate section.

## 4. The Role of ATR DDR in the Molecular Pathogenesis of HPV

### 4.1. A Brief Overview of DDR

**Figure 3:**
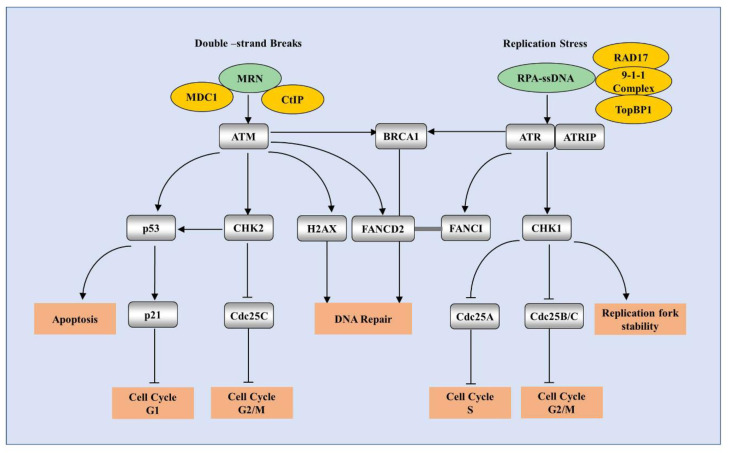
The schematic overview of the classical ATM and ATR DDR. The ATM/CHK2 pathway is activated by double-strand breaks, while ATR/CHK1 pathway is induced by single-strand breaks or replication stress. Activated ATM or ATR phosphorylates critical targets such as p53, CHK2, and CHK1, which are essential for regulating cell cycle checkpoints. Activated p53 leads to G1-phase cell cycle arrest and induces apoptosis. Phosphorylation of Cdc25 mediated by CHK2 and CHK1 results in cell cycle arrest in either S phase or G2/M phase. FANCD2 and FANCI, as the key components of the Fanconi anemia (FA) pathway, can be activated by ATM or ATR. In addition, activation of the ATM and ATR pathways play critical roles in regulation of DNA repair, apoptosis, and replication fork stability.

DDR is a complex signaling network directed to maintain cell preservation by regulating multiple pathways of DNA repair, cell-cycle arrest, apoptosis, etc. DDR is regulated by a family of phosphatidylinositol 3-kinase (PI3K)-related kinase (PIKKs), including ATM, ATR, and DNA-dependent protein kinase (DNA-PK) [5]. ATM is recruited to double-stranded breaks (DSBs) through the MRE11-RAD50-NBS1 (MRN) complex [66]. ATR is recruited to RPA-coated single-stranded DNA (ssDNA) in response to DNA replication stress [67], whereas DNA-PK is activated by Ku-bound DSB ends and participates DNA repair [68]. Three mechanisms have been shown to repair DSBs: non-homologous end joining (NHEJ), microhomology-mediated end joining (MMEJ) and homologous recombination (HR) [69,70]. ATM and ATR are two major kinases of HR, while DNA-PK works for NHEJ. The classical pathways of ATM and ATR DDR are summarized in Figure 3.

HPV requires activation of DDR for its life cycle [71]. First, HPV has to evade host surveillance during its replication synchronized with host genomes. For this purpose, HPV hijacks host DDR pathways to modulate cell cycle progression, DNA repair, and host transformation [72]. Second, HPV might create aberrant comprehensive DNA structures such as the “onion skin”-like structures during its DNA replication [71], which facilitates activation of DDR and in turn recruits DDR proteins to resolve the DNA complex. Third, previous studies have shown that DDR activation is necessary for HPV genome amplification [60,73]. Next, we will briefly describe the roles of ATM DDR in HPV productive replication, before we focus on the studies of the ATR pathway in the HPV life cycle.

### 4.2. Activation of the ATM Pathway is Necessary for HPV Productive Replication

ATM is one of the main kinases involved in HR-mediated DNA repair. Once recruited to sites of DSBs by the MRN complex, activated ATM promotes a series of coordinated cellular events such as checkpoint activation, DNA repair, and apoptosis [74]. The substrates of ATM include CHK2, p53, structural maintenance of chromosome1 (SMC1), Fanconi anemia group D2 protein (FANCD2), etc. [5]. Among them, CHK2 promotes G2/M phase arrest by negatively regulating Cdc25C on serine-216 [75]. The tumor suppressor p53 can be phosphorylated by ATM [76], which in turn activates its transcriptional activities to regulate cell-cycle arrest as well as apoptosis in response to DNA damage [77]. SMC1 serves as a downstream effector in the ATM/NBS1 branch, and phosphorylation of SMC1 by ATM is necessary for activating the S-phase checkpoint [78]. As a key component of the Fanconi anemia (FA) pathway, FANCD2 can be phosphorylated on serine 222 by ATM kinase, leading to activation of the S-phase checkpoint [79] and contribution to DNA repair by cooperation with BRCA1 [80]. Proposed as another downstream factor of ATM kinase [81], FANCI forms a complex with FANCD2 and facilitates DNA repair [82]. In addition, ATM is responsible for phosphorylation of NBS1 [83], BRCA1 [84], and the endonuclease CtIP [85], and histone H2AX [86], resulting in HR repair.

Because of their significant roles in HR repair, ATM has been studied for differentiation-dependent life cycle of HPV. Previous studies have shown that ATM is activated by HPV and that ATM is necessary for HPV genome amplification, but not genome maintenance or basal viral genome replication [73]. This activation can be regulated by the HPV E7 protein, and mutations of E7’s HDAC (histone deacetylase) or Rb-binding domains result in loss of phosphorylated ATM [73]. E7 also promotes activation of the STAT-5 signaling to facilitate ATM phosphorylation [60]. HPV E1 helicase can independently activate the ATM pathway in the nucleus, and nuclear export of E1 might suppress this activation [87]. In addition, E2 helps to E1 recruitment to the viral origin of replication, which leads to formation of nuclear foci that represent HPV DNA replication factories [88,89]. Co-expression of E1 and E2 facilitates loading of DDR factors to these nuclear foci, such as the MRN complex, ATM and CHK2 [88,90]. 

These above works reflect the complexity of how HPV activates DDR and raised another important issue of how HPV-infected cells are capable of proliferating in their maintenance phase while allowing induction of DDR that usually couples with the apoptotic signaling. This contradiction could be partly explained by behavior of E2. Presence of nuclear E2 reduces E1’s capabilities to induce DDR as well as activation of the ATM pathway [87,88]. Moreover, while HPV E7 largely promotes cell cycle progression and induces genomic instability that magnifies apoptotic signals, E6 prevents the infected cells from cell cycle exit or progress to apoptosis [91]. The effect of E6 in apoptosis inhibition relies on its abilities of promoting p53 degradation, decreasing levels of pro-apoptotic proteins such as Bak and Bax, and preventing the release of the apoptosis-inducing factor (AIF) from the mitochondria and the genomic DNA fragmentation [92,93,94].

The upstream factors of ATM activation, such as NBS1 and RAD50, can be activated by HPV [95]. Another ATM activation responsible enzyme, namely acetyltransferase Tip60 (Tat-interactive protein), can be induced by HPV, but not by either E6 or E7 [96,97], indicating that other viral proteins might participate this process. The downstream signaling molecules of ATM activation, such as the cohesion protein SMC1, p38, and FANCD2, are also important regulators for the HPV life cycle. SMC1 can be recruited to nuclear foci along with γ-H2AX, and further form a complex with DNA insulator protein CTCF to regulate HPV genome amplification [98]. The p38 MAPK pathway repairs damage to DNA to help regulate the HPV life cycle [99]. Finally, FANCD2 can contribute to regulation of the HPV genome maintenance [100], independently of ATM that does not directly regulate episome maintenance.

### 4.3. The Fanconi Anemia (FA) Pathway is Associated with HPV-Related Diseases

Fanconi anemia (FA) is a rare chromosome disorder syndrome characterized by genomic instability. FA patients have been confirmed to be susceptible to HPV-related head and neck squamous cell carcinoma (HNSCC) and skin tumors [101]. The FANC family includes ubiquitin ligase (FANCL), monoubiquitinated protein (FANCD2), breast/ovarian cancer susceptibility protein (FANCD1/BRCA2) and so on [101], which have been confirmed to be related to DNA damage repair.

The FA pathway can be activated by HPV [100]. Previous studies have shown that the expression of high-risk E7 activates the FA pathway, leading to chromosome instability in FA cells [102]. However, the roles of the FA pathway in HPV replication is a matter of debate. Contrasted with the FANCD2 findings described above, other studies showed that disruption of FA increases the amplification of the HPV genome in differentiated cells [103], which might explain why FA patients are more likely to induce HPV-related malignant tumors. Consistently, deletion of FANCA contributes to the accumulation of E7 protein and stimulates the growth of HPV cells [104]. In addition, loss of activities of the FA pathway is associated with the increasing incidence of HPV-induced carcinogenesis [105]. The difference between these studies may be attributed to distinction in experimental approaches. Further studies still need to be performed to clarify the roles of the FA pathway in HPV life cycle and how HPV regulates the FA pathway. Recent studies showed that the ATR–CHK1 pathway was another mechanism responsible for the activation of the FA pathway [106].

### 4.4. The ATR Pathway is Activated by HPV and Required for Efficient Viral Replication

ATR is activated in response to SSBs as well as abnormal DNA structures that arise as a result of stalled or collapsed replication forks [107]. The activation is initiated by the presence of co-factors such as replication protein A (RPA) and ATR interacting protein (ATRIP). The RPA-coated ssDNA complex can be bound to ATRIP, which facilitates the ATR–ATRIP complex to localize to sites of DNA damage [67]. Besides ATRIP, ATR activation requires the presence of regulatory partners such as RAD17 and the 9-1-1 complex (RAD9-RAD1-HUS1). In addition, Topoisomerase II-binding protein 1 (TopBP1) can be recruited by the 9-1-1 complex to trigger ATR activation [108]. Recent studies have shown that another replication stress response protein ETAA1 can bind to RPA and regulate activation of the ATR–ATRIP complex for maintenance of the genome stability [109]. Once activated, ATR phosphorylates many substrates, which consequently contribute to cell cycle arrest, DNA repair, or apoptosis [6]. As a key effector of the ATR pathway, CHK1 phosphorylates Cdc25A, Cdc25B, and Cdc25C to regulate cell cycle transitions by manipulating the cyclin-dependent kinases [110]. Phosphorylation of MCM and RPA by ATR contributes to maintain replication fork stability [111]. Another downstream target shared between ATR/ATM is tumor suppressor p53, which can be activated under genotoxic stress and induce cell apoptosis [112]. BRCA1 is phosphorylated by ATR on Serine 1423 in response to DNA replication stress [113]. In addition, ATR is essential for activating functions of multiple FA proteins such as FANCM [114], FANCA [115], FANCD2 [116], and FANCI [117]. 

While the critical roles of ATM DDR have been shown for HPV life cycle, increasing evidence has shown that activation of the ATR pathway is equally important to, if not more than, ATM DDR during HPV life cycle. Similar to what it does to the ATM pathway, HPV can induce activation of the ATR pathway and several HPV viral proteins can be responsible for this. For example, Both E1 and E2 can bind to RPA, which facilitates the localization of the ATR–ATRIP complex to DNA damage sites [118]. HPV18 E1 induces accumulation of ATRIP and TopBP1 in viral replication centers [119]. HPV E2 interacts with TopBP1 and mutation on E2’s BRCT (BRCA1 C Terminus) domains that bind to TopBP1 disrupts initiation of HPV DNA replication [120,121]. TopBP1 is also localized at E1-E2 replication complexes without exogenous genotoxic stress [122], providing the feasibility of easy recognition of other DDR factors associated with TopBP1 to aberrant DNA structures that are generated during viral DNA replication. Moreover, E7 induces STAT-5 phosphorylation to increase TopBP1 levels to facilitate ATR activation [8]. E7 has also been shown to increase turnover of claspin for mitotic entry [123]. In addition, E6 expression results in sustained CHK1 phosphorylation upon carcinogen-induced DNA damage [124]. Of note, CHK1 can feedback to phosphorylate HPV E6 PDZ binding motif, enhancing E6’s ability of repressing p53 transcriptional activities [125]. These works indicate that the HPV life cycle requires the activation of ATR DDR.

**Figure 4:**
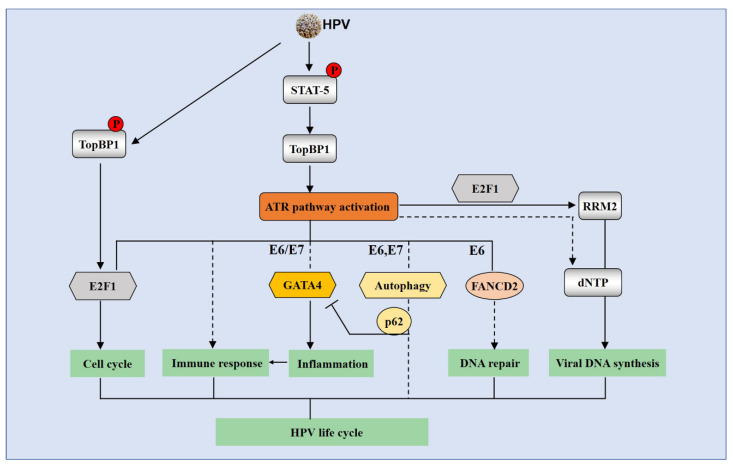
The ATR signaling pathway is involved in the HPV life cycle. HPV phosphorylates STAT-5 in the absence of exogenous DNA damage agents to increase TopBP1 levels, which activate the ATR signaling pathway. HPV also phosphorylates TopBP1 to regulate E2F1 transcriptional activities, which is important for HPV replication. ATR activation is necessary for HPV genomes replication through various mechanisms. The ATR/CHK1 DDR can regulate cell cycle arrest through E2F1 in HPV-positive cells. ATR possibly modulates immune response by GATA4-driven inflammation. ATR is also suggested to be responsible for induction of autophagy, which correlates with the GATA4 signaling via autophagy cargo protein p62. The ATR/CHK1 pathway can act with RRM2 to promote HPV DNA synthesis. The solid arrow means positive regulation, whereas the blocking arrow signifies inhibition. The dotted line represents that the role of the pathway remains uncertain.

Several studies have shown that activation of the ATR pathway is critical for HPV genome maintenance and amplification (Figure 4). Hong et al. have reported that ATR plays a key role in differentiation-dependent HPV genome amplification [8]. Inhibition of the ATR pathway by TopBP1 knockdown, ATR inhibitors, or CHK1 inhibitors suppresses HPV genome maintenance and amplification [7,8,53]. To keep the infected cells proliferative during this process, HPV induces ATR activation and CHK1 enhances E6’s abilities to abolish apoptosis via suppressing p53 transcriptional activities [125]. Furthermore, the fact that HPV E7 induces TopBP1-dependent ATR activation in E7-expressing cells raises the question of how E7 keeps the cells proliferative even without the help of E6 while inducing ATR activation that is strongly associated with replication stress and apoptosis. One key player could be the E2F proteins that function along with pRb in cell cycle regulation. HPV E7 is capable of binding to pRb and disassociating E2F proteins from pRb to drive cell cycle progression [126]. In addition, E7 induces TopBP1 expression and transcriptional activities to increase E2F1 levels [53]. The study of Moody Lab also independently showed that E2F1 can be activated by the ATR pathway in HPV-positive cells [127]. 

ATR activation might play other functions than DDR to contribute to HPV life cycle several studies have been deployed to investigate its downstream events, such as transcriptional regulation, autophagy, and DNA synthesis (Figure 4), which suggests that HPV uses ATR activation as a double sword to regulate not only DDR, but other protective mechanisms. Inhibition of the upstream factor TopBP1 reduces expression of E2F1 as well as p73 in HPV-positive cells [53], which consequently regulate expression of the genes related to cell cycle and differentiation [53] as well as RNA-binding proteins that control mRNA programming [128]. Phosphorylated by AKT [129], TopBP1 negatively regulates expression of many interferon-stimulated genes such as IFN-κ and other inflammatory cytokines such as IL-6 and IL-8 [53]. Recent studies have shown that IFN-κ can inhibit HPV transcription [130]. Consistently, ATR has been reported to modulate the immune microenvironment [131]. Along with this idea, the Elledge group [132] identified that suppression of the ATR signaling by inhibitors in HPV E6/E7-expressing cells leads to a reduction of GATA4-dependent inflammation, which is proposed to be through autophagy cargo protein p62, which might be suppressed by ATR. This observation is interesting and raised an important question of whether HPV activates ATR to regulate inflammatory response. Our data show that the inhibition of ATR activation by silencing TopBP1 [8] or ATR (unpublished data) results in a significant increase of expression of inflammatory genes in HPV-positive cells, which is different from the Elledge group’s studies in fibroblasts indicating that ATR induces inflammation. This may be due to the difference of how ATR regulates the p62 signaling in different cell types and should be addressed in future studies. Our observation supports the idea that HPV utilizes ATR to inhibit the inflammatory response in HPV-positive keratinocytes and that this modification benefits HPV-infected cells escaping from potential senescence signals associated with inflammation.

P62 is an autophagy cargo protein that targets proteins for selective autophagy [133]. Autophagy primarily acts as a protective mechanism for cell survival in response to various stresses. It is not well studied whether ATR DDR suppresses autophagy while inducing genome instability. A recent study identified that autophagy can be induced by the ATR/CHK1 signaling in a RhoB-dependent manner [134]. The role of autophagy in the HPV life cycle is still cloudy. HPV16 E7 has been shown to upregulate autophagy marker LC3B [135]. E6 is capable of activating autophagy by suppressing the mTOR (mammalian target of rapamycin) signaling [136]. The combination of E6 and E7 can increase the autophagosome cycle rate via ATG9B(Autophagy-related protein 9B) and LAMP1 (Lysosome-associated membrane glycoprotein 1) [137]. However, other studies reported that deletion of HPV16 early genes activates autophagy and that HPV16 cellular entry requires inhibition of autophagy [138]. It is not yet clear whether ATR regulates autophagy during HPV infection. According to Elledge’s findings, ATR should decrease p62 expression and suppress autophagy, which is consistent with Ozbun group’s observations described above. Further studies should be performed by using ATR knockdown cells to address the effects directly. 

In addition, HPV utilizes ATR in other aspects such as DNA synthesis. For example, HPV acts through the ATR/CHK1/E2F1 pathway to regulate RRM2, a component of the ribonucleotide reductase (RNR) complex, to mediate HPV viral DNA synthesis [127]. HPV increases RRM2 expression and results in accumulation of dNTP pools. Silencing RRM2 suppresses HPV replication, indicating that HPV utilizes RRM2 to provide enough supplies for its replication. It is not clear whether the effect of RRM2 on viral replication is dependent on replication timing. Other mechanisms of ATR-regulated dNTP accumulation are also not excluded. 

Different from α HPV types described above, β HPV types suppress ATR activation. For example, HPV5 E6 interacts with p300 to disrupt activation of the ATR pathway, resulting in increasing UVB-induced DSBs [139]. Consistently, Hufbauer M group [140] also observed that HPV8 E6 decreases phosphorylation of ATR and CHK1. The difference of the effects on ATR activation between high-risk and β HPV types may come from different functions and structures of E6 and E7 proteins. β HPV E6 neither targets p53 for degradation nor binds to PDZ proteins, and β HPV E7 fails to affect RB levels, which may result in a reduction of the ATR signaling.

In addition to HPV, other viruses have evolved to utilize the ATR pathway to accomplish their life cycles. For instance, HSV-1 and human parvovirus B19 (B19V) induce ATR activation for their DNA replication [141,142]. In contrast, the Zika virus (ZIKV) inhibits activation of the ATR/Chk1 pathway to benefit its viral replication [143]. Adenovirus can reduce ATR phosphorylation or promote TopBP1 degradation to contribute to viral production efficiency [144,145]. The Epstein–Barr virus (EBV) infection promotes Claspin loss to impair ATR activation, leading to cell proliferation [146]. Additionally, EBV lytic infection induces ATM activation and ATM inhibition leads to suppression of EBV DNA replication [147]. EBV can also downregulate ATM expression via miRNAs to promote tumorigenesis of nasopharyngeal carcinoma [148,149]. These suggest that the effects of EBV on ATM DDR are complicated and might depend on the levels of DDR activation. Taken together, these data provide insights into the diverse effects of the ATR pathway in the life cycle of different DNA/RNA viruses. 

## 5. Potential Therapeutic Prospects of ATR DDR Inhibitors Combined with Traditional Therapy for HPV-Associated Cancer

ATR has been investigated for its potential as a novel target for anti-HPV treatment. Treatment of HPV-positive cells with VE-822 triggers DNA breaks and the fragmentation of viral episomes [150], which is consistent with our previous findings that suppression of ATR activation by either VE-822 or CHK1 inhibitor UCN-01 dampens HPV genome amplification [8,127]. Another CHK1 inhibitor, MK-8776, has been shown to reduce HPV DNA amplification and to induce apoptosis in HPV-infected cells [151]. These studies with ATR inhibitors set a promising base for anti-HPV drug development. In addition, treatment of ATR inhibitors AZD6738 significantly increased the sensitivity of HPV-positive HNSCC cells and xenograft tumors to cisplatin [9]. Co-treatment with AZD6738 plus cisplatin improves the anti-tumor activities of HNSCC patients while reducing the adverse effects of cisplatin. Combination of CHK1 inhibitors and the EGFR inhibitor cetuximab, along with radiotherapy, can decrease proliferation of HPV-positive HNSCC cells, showing significant anti-tumor effects in vivo and in vitro [10]. Current studies about ATR/CHK1 inhibitors targeting HPV-related diseases are summarized in Table 1.

## 6. Summary and Outlook

HPV pathogenesis is tightly associated with its DNA replication. The productive replication of HPV requires activation of the host cellular DDR pathways, such as the ATM pathway, the FA pathway and the ATR pathway. We described in detail the importance of the ATR pathway in HPV DNA replication. High-risk HPVs induce ATR expression and phosphorylation through various mechanisms. Inhibition of the upstream and the downstream factors of the ATR pathway, as well as ATR itself, results in a significant reduction of HPV genome maintenance and amplification, suggesting a critical role of ATR in HPV life cycle. Recent findings have shown that ATR activation impacts cellular transcription regulation and viral DNA synthesis, which provide new insights into understanding the comprehensive molecular mechanisms of ATR activation for the HPV life cycle. HPV utilizes its viral proteins to tune the balance between ATR DDR and cell apoptosis caused by replication stress. Moreover, ATR activation by HPV induces non-DDR functions for the HPV life cycle. Further studies need to investigate more thoroughly how ATR and downstream pathway components regulate HPV DNA replication. In addition, we outlined the therapeutic prospects of current ATR DDR inhibitors for HPV-associated cancers combined with traditional therapies. With the growth of knowledge of ATR functions in HPV life cycle, the more efficient ATR DDR inhibitors will be expected to be developed to block the viral DNA replication, reduce HPV-induced malignancies, and eventually cure the patients that have been infected by HPV.

## Figures and Tables

**Table 1:** Current status of ATR/CHK1 inhibitors in HPV-associated diseases.

Approach	Name	Cancer	Treatment	Clinical Trials	References
ATR Inhibitors	VE-822 (VX-970)	HPV(+) cells	/	-	Nakahara T, J Virol. 2015
	AZD6738	HPV(+)HNSCC cells and patient-derived xenograft tumors	Radiation Cisplatin	-	Dillon MT, Clin Cancer Res. 2019 Leonard BC, Oral Oncol. 2019
	BEZ235	HPV(+)tonsillar and base of tongue squamous cell carcinoma, HPV anal carcinogenesis mouse model, HPV(+)HNSCC cells	FGFR inhibitor AZD4547, Radiation	-	Holzhauser S, Oncol Lett. 2019 Rademacher BL, Eur J Cancer Prev. 2019 Schötz U, Cancers (Basel). 2020
CHK1 Inhibitors	AZD7762	HPV(+)HNSCC cells	/	-	Ghasemi F, Oncotarget. 2018
	LY2603618	HPV(+)HNSCC cells	Wee1 inhibitor AZD1775 Radiation	-	Busch CJ, Radiother Oncol. 2017
	MK-8776	Cervical cancer cells HPV(+)HNSCC cells	Cisplatin PARP inhibitor niraparib Radiation	-	Banerjee NS, Int J Mol Sci. 2019 Molkentine JM, Int J Radiat Biol. 2020
	LY2606368	HPV(+)HNSCC cells	EGFR inhibitor cetuximab Radiation	Phase 1 NCT02555644	Zeng L, Mol Cancer Ther. 2017
	CCT244747	HPV(+)HNSCC cells	Radiation paclitaxel	-	Barker HE, Mol Cancer Ther. 2016

/ This kind of inhibitor is used alone in HPV diseases; - this kind of inhibitor remains in preclinical study.
